# Mendelian Randomization Study Investigating the Causal Relationship Between Thyroid Dysfunction and Cerebral Infarction

**DOI:** 10.1002/brb3.70188

**Published:** 2024-12-11

**Authors:** Letai Li, Jiajie Leng, Haibing Xiong, Zishan Deng, Meng Ye, Haiyan Wang, Xin Guo, Shi Zeng, Haofeng Xiong, Jianhong Huo

**Affiliations:** ^1^ The First Clinical College of Chongqing Medical University Chongqing China; ^2^ Department of Neurosurgery Banan Hospital of Chongqing Medical University Chongqing China; ^3^ Department of Cardiothoracic Surgery The First Affiliated Hospital of Chongqing Medical University Chongqing China; ^4^ Department of Neurosurgery Chengkou County People's Hospital Chongqing

**Keywords:** cerebral infarction, hyperthyroidism, Mendelian randomization analysis, thyroid dysfunction

## Abstract

**BACKGROUND:**

There is an association between thyroid dysfunction and cerebral infarction (CI), but the causality cannot be determined. A two‐sample two‐way Mendelian randomization (MR) study was conducted to assess the causal relationship between thyroid function and CI.

**METHODS:**

We selected single‐nucleotide polymorphisms (SNPs) associated with five phenotypes, including CI from the UK Biobank (*n* = 361,194), hyperthyroidism from the IEU Open GWAS database (*n* = 484,598), hypothyroidism from the IEU Open GWAS database (*n* = 473,703), normal thyroid‐stimulating hormone (TSH) (*n* = 271,040), and normal free thyroxine (FT4) (*n* = 119,120) from the Thyroidomics Consortium database. For the forward MR analysis, the exposures were hyperthyroidism, hypothyroidism, TSH, and FT4. The inverse variance weighted (IVW) method, weighted median (WM), and MR‐Egger revealed the causality with CI. For the reverse MR analysis, CI was regarded as the exposure, and four thyroid function phenotypes were the outcomes. The sensitivity and heterogeneity test was assessed using Cochran's Q test, MR‐Egger regression, and leave‐one‐out analysis.

**RESULTS:**

The MR analysis indicated that genetic susceptibility to hyperthyroidism increased the risk of CI (IVW‐OR = 1.070; 95% CI: 1.015–1.128; *p* = 0.003). In reverse MR, genetic susceptibility to RA is not associated with hyperthyroidism (IVW‐OR = 1.001; 95% CI: 1.000–1.001; *p* = 0.144). Any positive or reverse causal relationship between hypothyroidism, FT4, and TSH with CI could not be established. Sensitivity and heterogeneity test consolidated our findings.

**CONCLUSION:**

The causality between CI and hyperthyroidism demonstrated patients with hyperthyroidism have a risk of genetic variants for CI. In the future, further studies are needed to fully explore their mechanisms of action.

## Graphical abstract

A potential association between genetic susceptibility to thyroid dysfunction and cerebral infarction (CI) was investigated. The patients with hyperthyroidism may have a greater risk of CI in terms of genetic variation.

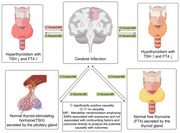

## 1 Background

Cerebral infarction (CI) is a prevalent neurological disorder that results in brain tissue damage due to an interruption of the blood supply to the brain. This is caused by either a sudden rupture or blockage of blood vessels in the brain, which prevents the brain from receiving sufficient oxygen and nutrients (Lo, Dalkara, and Moskowitz 2003). The global prevalence of CI is also quite high and is one of the leading causes of long‐term disability and death. The disease is characterized by the sudden onset of neurological deficits such as hemiparesis, aphasia, and visual impairment. Furthermore, CI can lead to cognitive impairment, affective disorders, and a decrease in daily living skills (W. Wang et al. 2017). Its occurrence is associated with a variety of genetic and environmental factors, including hypertension, diabetes mellitus, and hypercholesterolemia (Ellekjaer et al. 1992). However, because CI is a multifactorial disease, it is difficult for traditional epidemiologic studies to accurately differentiate the causal relationship between factors (Rutten‐Jacobs et al. 2018).

According to *The Lancet* guidelines, clinical hyperthyroidism was defined as thyroid‐stimulating hormone (TSH) below the normal range (0.4–4.0 mIU/L) and free thyroxine increased, whereas clinical hypothyroidism was defined as TSH above the normal range(0.4–4.0 mIU/L) and free thyroxine decreased (Chaker et al. 2024; Taylor et al. 2024). Thyroid dysfunction, especially hyperthyroidism, is likewise recognized to be associated with the onset and progression of CI. Changes in thyroid hormone levels play an important role in physiologic processes, and they can affect several aspects of vascular function, blood coagulation, and inflammatory responses, thus indirectly influencing the risk of CI (Surks et al. 2004). Although there is a substantial body of literature on the subject, including numerous case reports and systematic reviews, the evidence regarding the causal association between thyroid dysfunction and CI remains inconclusive. Further research is necessary to substantiate this relationship (Chaker et al. 2014; Bi et al. 2012; Ahmed and Tabet 2015; Ohba, Nakagawa, and Murakami 2011). The application of the two‐way two‐sample Mendelian randomization (MR) method provides a novel approach to investigating the causal role of genetic factors in CI.

It is acknowledged that traditional observational studies are susceptible to confounding factors, that randomized controlled trials are constrained by a variety of real‐world conditions that make them difficult to implement, and that inferences of causality are limited and unreliable. MR is a novel data analysis method for evaluating causal inferences in medical statistical studies. It employs genetic variants with robust correlations with exposure factors as instrumental variables (IVs) to assess the causal relationship between exposure factors and outcomes (Davey Smith and Hemani 2014). As genetic variants are present at birth and remain stable throughout the life cycle, results derived from MR analysis are less susceptible to causal inversion and confounding (Sekula et al. 2016). Accordingly, this study employed the potential causal relationship between hypothyroidism, hyperthyroidism, normal TSH, normal free thyroxine (FT4), and CI. Furthermore, the aim is to elucidate the interactions between the variables in question and to provide new insights into the underlying mechanisms.

## 2 Methodology

### 2.1 Research Design

**FIGURE 1:**
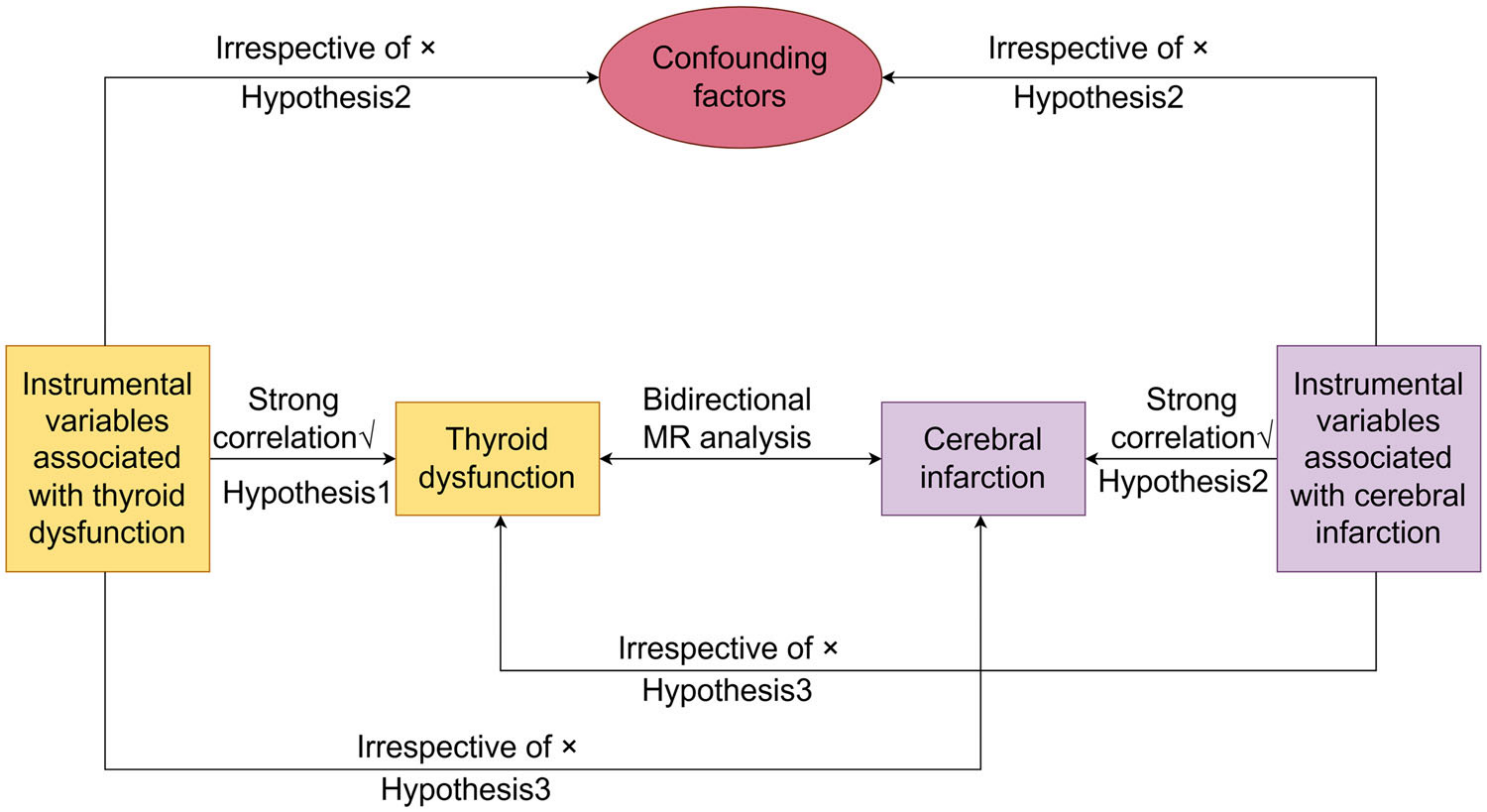
The flowchart about the bidirectional two‐sample Mendelian randomization research to explore the causal relationship between thyroid dysfunction and cerebral infarction.

In order to obtain reliable results from a MR analysis, it is essential that the IV in question meets three key assumptions (Hemani et al. 2018; Burgess, Butterworth, and Thompson 2013; Freuer, Linseisen, and Meisinger 2022; Burgess, Small, and Thompson 2017; Burgess et al. 2015). First, the IV and the exposure must be closely related. Second, the IV must be independent of any confounding factors that may affect the exposure and the outcome. Third, the IV must affect the outcome only through the exposure. Furthermore, this study adhered to the most recent guidelines for MR in epidemiological studies (STROBE‐MR) (Skrivankova et al. 2021). The study comprised the following core steps: the selection of genetic IVs associated with exposure using multiple MR methods, multiplicity assessment, and heterogeneity and sensitivity analyses. To examine the association between thyroid dysfunction and CI, a bidirectional two‐sample MR study was employed. To minimize bias due to population stratification and ethnic differences, samples were selected only from European populations. Figure 1 illustrates the flowchart of the MR research.

### 2.2 Data Sources

The data pertaining to genetic associations with CI were obtained from the UK Biobank (ukb‐d‐I63). This database comprises a total sample size of 361,194 individuals, including 2353 CI cases and 358,841 controls (Grama et al. 2020).

The GWAS data for hyperthyroidism in thyroid dysfunction were obtained from the IEU Open GWAS database (ebi‐a‐GCST90038636) (https://gwas.mrcieu.ac.uk/) with a sample size of 484,598 individuals, including 3731 cases of hyperthyroidism and 480,867 controls (T. Wang et al. 2024). The GWAS data for hypothyroidism were obtained from the IEU Open GWAS database (ebi‐a‐GCST90029022) (https://gwas.mrcieu.ac.uk/), including 473,703 total individuals.

Furthermore, the most recent data from the Thyroidomics Consortium 2024 (www.thyroidomics.com) for pooled levels of normal TSH and normal FT4 in individuals with normal thyroid function were employed, with sample sizes of 271,040 and 119,120 individuals, respectively (Weihs et al. 2023; Ellervik et al. 2024). Given the strong association between thyroid dysfunction and circulating TSH and FT4 levels, we selected the data pertaining to normal TSH and FT4 levels and conducted an MR analysis on them for the purpose of establishing a control group.

### 2.3 IV Selection

In order to fulfill the initial step of the initial hypothesis, namely the construction of the genetic IV, single‐nucleotide polymorphisms (SNPs) that are significantly associated with the exposure were identified based on the application of rigorous criteria (*p* < 5 × 10⁻⁸) and the fulfillment of independence requirements (*r* ^2^ < 0.001, kb = 10,000). However, in a reverse MR analysis with CI as the exposure, no SNP was found to be significantly associated with exposure at a *p*‐value of less than 5 × 10^−8^. Consequently, in the reverse MR analysis, the threshold for statistical significance was set at *p* < 5 × 10^−5^.

It was not deemed appropriate to utilize SNP proxies, and the minimum allele frequency (MAF) was set at 0.001. Furthermore, the effect alleles were harmonized between the exposure and outcome datasets, with all SNPs with palindromes and ambiguities excluded. To assess the strength of the IV, the F‐statistic value was calculated as *F* = *β* ^2^/se^2^. An *F* value greater than 10 indicates a low risk of weak IV bias and avoids weak tool bias (Burgess Thompson, and CRP CHD Genetics Collaboration 2011; Pierce, Ahsan, and Vanderweele 2011).

### 2.4 Statistical Analysis

In this study, three methods were employed to determine the causal relationship: inverse variance weighted (IVW) and MR‐Egger, weighted median (WM). In terms of efficacy, IVW is the method with the strongest statistical credentials, particularly when all instruments employed in an analysis have been validated (Burgess et al. 2019). In establishing causality, significant findings from IVW analyses were taken into account. Additionally, the results from WM and MR‐Egger analyses were found to align with those from IVW in the same direction.

A sensitivity analysis was conducted using the MR‐Egger intercept to ascertain the extent of pleiotropy. Intercept values approaching 0 and *p*‐values exceeding 0.05 indicate the absence of horizontal pleiotropy (Chen et al. 2022). Subsequently, Cochran's Q‐test was employed to quantify heterogeneity in IVW estimates, with a *p*‐value > 0.05 indicating the absence of heterogeneity (Venkatesh et al. 2022). In order to assess the robustness of the results, we also conducted leave‐one‐out analyses to examine the impact of individual SNPs on the overall causal effect (Cheng et al. 2022). Funnel plots were employed to assess the symmetry of the selected SNPs, forest plots were utilized to assess the reliability and heterogeneity of chance estimates, and scatter plots were employed to visualize the effect relationship between exposure and outcome (Hartwig, Davey, and Bowden 2017; Bowden et al. 2019). In a similar manner, the reverse analysis employed the identical methodology as previously described, utilizing the set of associated SNPs with CIs to investigate the causal effects between the latter and thyroid function, including hypothyroidism, hyperthyroidism, FT4, and TSH. The entire analysis was performed using RStudio (version 4.3.0), with the “TwoSampleMR” package employed.

## 3 Results

### 3.1 Selection of IV

In forward analysis, we obtained 19, 129, 65, and 153 IVs from hyperthyroidism, hypothyroidism, FT4, and TSH, respectively, independent of linkage disequilibrium (LD). In reverse analyses, 110, 110, 93, and 95 CI‐associated SNPs were selected as IVs for CI. *F*‐statistic values greater than 10 were obtained for each of the selected IVs, suggesting that weak IV bias is unlikely to be present. The SNPs were selected as IVs in the reverse analyses. Tables S2–S9 provide a list of the exposure information for the SNPs.

### 3.2 Forward MR and Sensitive Analysis

As demonstrated in the supplementary table, following the removal of 2, 6, 2, and 2 palindromic or heterozygous SNPs associated with hyperthyroidism, hypothyroidism, TSH, and FT4, respectively, a final set of 17, 123, 151, and 63 SNPs was obtained for each exposure in the analysis.

**FIGURE 2:**
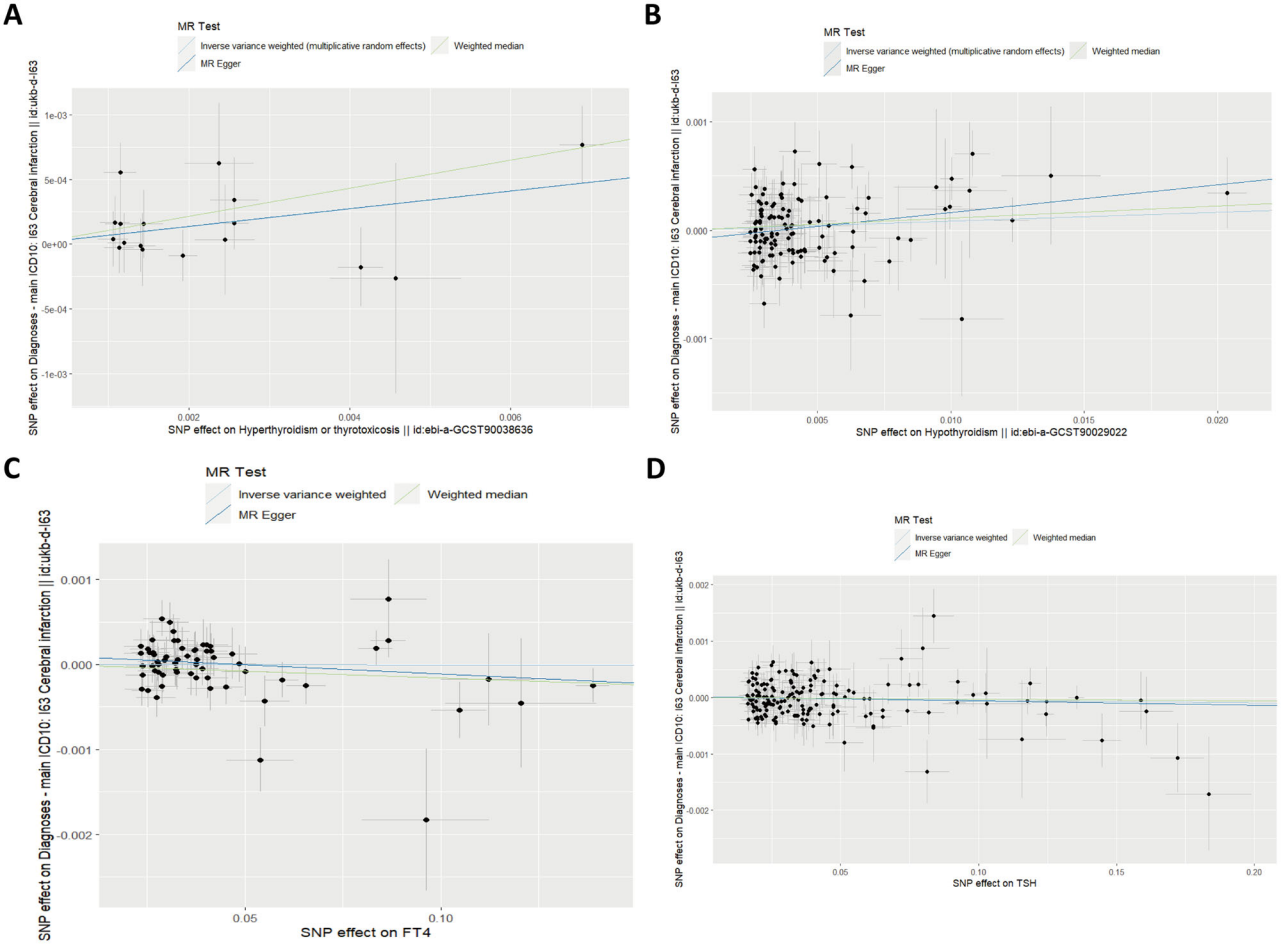
Scatter plot analysis of the causal relationship between thyroid function and cerebral infarction in the initial MR analysis was performed using IVW, MR‐Egger regression, weighted median, simple model, and weighted model. The slope of each line corresponds to the MR effect estimated by each method. (A) Hyperthyroidism, (B) hypothyroidism, (C) FT4, and (D) TSH. ivw, inverse variance weighting; MR, Mendelian randomization; MR‐Egger, MR‐Egger regression.

The IVW analysis demonstrated that there is a significantly elevated risk of cardiovascular incidents in individuals with hyperthyroidism (OR = 1.070, 95% CI: 1.015–1.128, *p* = 0.003). There was no significant causal association between hypothyroidism, TSH, and FT4 and RA, as demonstrated in Table 1. Scatter plots of SNP effect sizes for each phenotype in the forward analysis are presented in Figure 2. No horizontal pleiotropy was observed for all phenotypes (MR‐Egger intercept, *p* > 0.05). Other SNPs associated with exposure exhibited no heterogeneity following the removal of palindromic SNPs. However, heterogeneity was observed in the final analysis of SNPs associated with TSH (Table 3). Consequently, we applied the IVW random effects method for the final analysis of TSH and the fixed effects method for the remaining exposures.

**TABLE 1:** MR results for the relationship between thyroid function on CI.

Exposure	Outcome	SNPs	OR	95% CI	*p*‐Value
Hyperthyroidism					
IVW	CI	17	1.070	1.015–1.128	0.003
MR‐Egger	CI	17	1.071	0.976–1.174	0.170
Weighted median	CI	17	1.114	1.038–1.197	0.005
Hypothyroidism					
IVW	CI	123	1.008	0.998–1.018	0.096
MR‐Egger	CI	123	1.025	1.005–1.047	0.015
Weighted median	CI	123	1.011	0.996–1.026	0.162
FT4					
IVW	CI	63	1.000	0.999–1.001	0.930
MR‐Egger	CI	63	0.998	0.995–1.000	0.130
Weighted median	CI	63	0.999	0.996–1.000	0.130
TSH					
IVW	CI	151	1.000	0.999–1.000	0.320
MR‐Egger	CI	151	0.999	0.998–1.001	0.380
Weighted median	CI	151	1.000	0.998–1.001	0.670

*Note*: All statistical tests were two‐sided. *p* < 0.05 was considered significant.

Abbreviations: 95% CI, confidence interval; CI, cerebral infarction; FT4, free thyroxine; IVW, inverse variance weighted; nSNP, number of single‐nucleotide polymorphisms; OR, odds ratio; TSH, thyroid‐stimulating hormone.

### 3.3 Reverse MR and Sensitive Analysis

Once the palindromes and heterozygous SNPs had been removed, 101, 106, 85, and 87 SNPs were included in the final analyses for hyperthyroidism, hypothyroidism, TSH, and FT4, respectively (Table S6). Consequently, the reverse MR demonstrated that the presumed causal associations between CI and hyperthyroidism, CI and hypothyroidism, CI and TSH, and CI and FT4 were not significant when all three analytical methods were employed (Table 2) (*p* > 0.05). These results indicated that there were no statistically significant causal relationships between CI and hyperthyroidism, hypothyroidism, TSH, and FT4.

**TABLE 2:** MR results for the relationship between CI on thyroid function.

Exposure	Outcome	SNPs	OR	95% CI	*p*‐Value
CI					
IVW	Hyperthyroidism	101	0.998	0.931–1.071	0.977
MR‐Egger	Hyperthyroidism	101	0.908	0.786–1.049	0.197
Weighted median	Hyperthyroidism	101	0.957	0.901–1.016	0.151
CI					
IVW	Hypothyroidism	106	1.039	0.862–1.254	0.682
MR‐Egger	Hypothyroidism	106	0.987	0.674–1.444	0.946
Weighted median	Hypothyroidism	106	1.059	0.914–1.227	0.444
CI					
IVW	FT4	85	0.889	0.245–3.227	0.858
MR‐Egger	FT4	85	0.133	0.009–1.952	0.145
Weighted median	FT4	85	1.816	0.332–9.932	0.491
CI					
IVW	TSH	87	0.655	0.296–1.447	0.296
MR‐Egger	TSH	87	0.313	0.060–1.632	0.171
Weighted median	TSH	87	0.547	0.185–1.620	0.276

*Note*: All statistical tests were two‐sided. *p* < 0.05 was considered significant.

Abbreviations: 95% CI, confidence interval; CI, cerebral infarction; FT4, free thyroxine; IVW, inverse variance weighted; nSNP, number of single‐nucleotide polymorphisms; OR, odds ratio; TSH, thyroid‐stimulating hormone.

**FIGURE 3:**
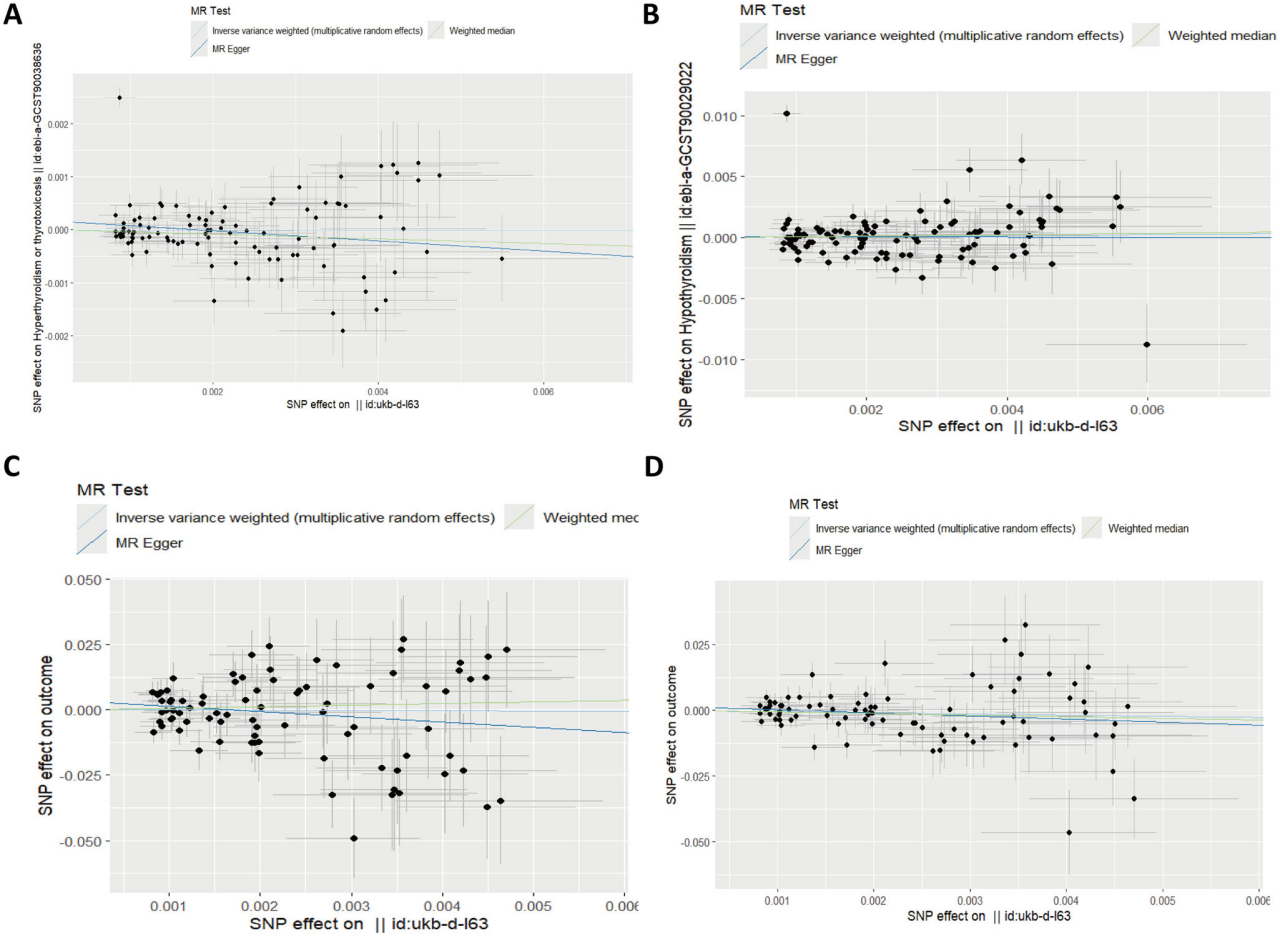
Reverse scatter plot analysis of the causal relationship between thyroid function and cerebral infarction in the initial MR analysis was performed using IVW, MR‐Egger regression, weighted median, simple model, and weighted model. The slope of each line corresponds to the MR effect estimated by each method. (A) Hyperthyroidism, (B) hypothyroidism, (C) FT4, and (D) TSH. ivw, inverse variance weighting; MR, Mendelian randomization; MR‐Egger, MR‐Egger regression.

Figure 3 presented scatter plots of SNP effect sizes for each phenotype in the reverse analysis. The MR‐Egger intercept test revealed no evidence of horizontal pleiotropy. A significant degree of heterogeneity was observed in TSH to CI, CI to hyperthyroidism, CI to hypothyroidism, and CI to FT4 (Table 3), which were assessed using a random‐effects model. The inverse‐variance weighted fixed‐effects model was employed to analyze hyperthyroidism and FT4. Furthermore, the “leave‐one‐out” analysis and graphical representation of the results demonstrate the robustness of the MR findings. The forest, leave‐one‐out sensitivity, and funnel plots of this study are presented in the Figures S1–S6.

**TABLE 3:** The heterogeneity and sensitivity of CI and thyroid function.

Exposure–Outcome	nSNP	MR‐Egger intercept		Cochran's heterogeneity			
		Intercept value	*p*	IVW‐Q value	*p* (IVW)	Egger‐Q value	*p* (Egger)
Hyperthyroidism‐CI	17	2.2315E‐07	0.9983415	12.10528	0.7367013	12.10527	0.6710437
Hypothyroidism‐CI	123	−9.06199E−05	0.06252803	149.99	0.04339131	145.7336	0.06237767
FT4‐CI	63	0.000106367	0.09659879	71.63239	0.1886014	68.43723	0.2396028
TSH‐CI	151	1.45213E‐05	0.701968	210.1263	0.00754777	209.9359	0.006673896
CI‐Hyperthyroidism	101	0.000167923	0.1467224	289.9484	< 0.000001	289.9484	< 0.000001
CI‐Hypothyroidism	106	9.74E‐05	0.7576465	382.0275	< 0.000001	381.6763	< 0.000001
CI‐FT4	85	0.003273793	0.1188957	114.0645	0.01620487	110.7514	0.0226178
CI‐TSH	87	0.001293683	0.3205917	98.20636	0.1735321	97.0665	0.1747524

Abbreviations: CI, cerebral infarction; FT4, free thyroxine; IVW, inverse variance‐weighted; MR, Mendelian randomization; nSNP, number of single‐nucleotide polymorphisms; TSH, thyroid‐stimulating hormone.

## 4 Discussion

This bidirectional two‐sample MR study provided novel evidence supporting a bidirectional causal relationship between thyroid dysfunction and CI. A genetic predisposition to hyperthyroidism is correlated with an elevated risk of CI. However, a genetic predisposition to CI is not linked with an increased risk of hyperthyroidism. Moreover, there was no evidence to indicate a positive or negative causal relationship between hypothyroidism, FT4, and TSH, with CI.

The results of our study aligned with those of previous observational studies. The presence of hyperthyroidism is a known risk factor for CI. The results of a five‐year follow‐up study on the incidence and risk factors of ischemic stroke among 3,176 individuals with diagnosed hyperthyroidism and 25,408 control subjects aged 18–44 revealed that the prevalence of ischemic stroke in the hyperthyroid group (1.0%) was higher than in the control group (0.6%) (HR = 1.44, 95% CI: 1.02–2.12, *p* = 0.038) (Sheu et al. 2010). Compared with previous studies, our MR results had less confounding factors, and the causal direction between hyperthyroidism and CI was clearly defined on the basis of genetic variation. A further study of 59,021 patients with hyperthyroidism and 1,180,420 controls, which was similarly conducted, demonstrated that hyperthyroidism increased the risk of ischemic stroke, independent of other risk factors. The HR value was calculated to be 1.12, with the 95% confidence interval being 1.04 to 1.20 (Kim et al. 2020; Sproviero et al. 2021). Although observational studies have been widely employed in the clinical setting for the initial identification of causality, reverse causality and potential confounders inevitably exist, rendering them less credible. The MR method, which employs genetic variation as an instrumental variable, offers a solution to the aforementioned difficulties, resulting in more credible findings of causality (Zheng et al. 2017; Smith et al. 2007; Smith and Ebrahim 2004). In comparison to the preceding conversational study, our MR findings were distinguished by a substantial sample population, multicenter data, the most recent data, and high efficiency.

Hyperthyroidism causing CI can be attributed to several mechanisms. In general, it may be related to mechanisms, including cerebral atherosclerosis, hypercoagulability and thrombosis, ischemia‐reperfusion injury due to hypermetabolic syndrome, atrial fibrillation, etc. First, thyroid hormones are able to influence vascular function. Elevated thyroid hormone levels may result in vasoconstriction, increased vascular resistance, and cerebral atherosclerosis (Pappan et al. 2022). Intracranial atherosclerotic disease has emerged as an important pathogenesis of CI and an associated high risk of CI (Al Kasab et al. 2018). Second, alterations in thyroid hormone levels also contributed to hypercoagulability and thrombosis in the cerebral artery. Elevated thyroid hormone levels may promote platelet aggregation and thrombosis, thereby increasing the risk of CI (Bano et al. 2019). The vWF values were significantly higher in hyperthyroid patients than in hypothyroid patients and normal thyroid controls (Antonijevic et al. 2024). Elevated levels of vWF, FVIII, and fibrinogen, as well as reduced fibrinolytic activity and reduced fibrinogen levels, contributed to the hypercoagulable state of patients with hyperthyroidism and predispose them to thromboembolism and vascular disease (Thompson et al. 1995). Third, hyperthyroidism can result in the development of atrial fibrillation, which in turn can lead to CI. Hyperthyroidism can result in sympathetic excitation, which in turn can lead to atrial remodeling (Antonijevic et al. 2024). Various studies have shown that atrial fibrillation is a significantly increased risk in patients with hyperthyroidism (16%–60% of patients complicated) and that such patients are prone to cardioembolic strokes (Inoue Kosuke et al. 2023). In addition, the high metabolic syndrome, which is a state of increased metabolic rate, can place a significant burden on CI. Several epidemiological studies have shown that the impact of hyperthyroidism on CI prognosis and severity is worsened (Chaker et al. 2016). The reason behind this association may be the effect of thyroid hormones on the phenomenon of ischemia/reperfusion. When in excess, THs can exacerbate the effects of the sympathetic nervous system (Mokhtari et al. 2017), leading to a dangerous hypermetabolic state characterized by increased production of reactive oxygen species and free radicals, resulting in cytotoxicity (Aslan et al. 2011).

In addition, having found a causal relationship between hyperthyroidism and CI, in order to identify the causal relationship between subclinical hyperthyroidism (normal FT4 combined with a single low TSH level) and CI, the present study included people with normal thyroid function in the Thyroid Consortium for their normal FT4 and TSH levels. We found that single levels of FT4 and TSH in people without thyroid dysfunction do not have a significant causal effect on CI. This suggested that the development of CI due to hyperthyroidism is the result of a combination of both hormonal abnormalities. These control group findings consolidated the above findings and highlighted the complexity of the mechanisms involved, which urgently need to be further explored.

It should be noted that the present study was subject to a number of limitations. First, the limited number of SNPs in the database prevented a comprehensive examination of the relationships between high levels of TSH and low levels of TSH, which are considered within the normal TSH range in our study of bidirectional causality with CI. Second, the database did not include a category for hyperthyroidism. Consequently, further investigation into the association between various hyperthyroidism and CI was not possible. Furthermore, the study population was exclusively comprised of individuals with European ancestry. It remains unclear whether the findings can be extrapolated beyond this specific group and whether they may not necessarily be applicable to other populations.

## 5 Conclusion

In conclusion, the results of our study corroborate the hypothesis that hyperthyroidism is a risk factor for CI. We found that hypothyroidism, FT4, and TSH levels are not associated with CI, which provides novel insights for the prevention and treatment of CI. In addition, it is essential to gain further insight into the potential underlying mechanisms of thyroid dysfunction and cognitive impairment. This can be achieved through large‐scale randomized controlled trials and scientific animal experiments in order to validate the observed association.

## Author Contributions

**Letai Li**: writing–review and editing, writing–original draft, conceptualization, methodology. **Jiajie Leng**: investigation, writing–original draft. **Haibing Xiong**: writing–original draft, writing–review and editing, funding acquisition, resources. **Zishan Deng**: conceptualization, methodology. **Meng Ye**: formal analysis, data curation. **Haiyan Wang**: visualization. **Xin Guo**: methodology. **Shi Zeng**: supervision, data curation. **Haofeng Xiong**: supervision. **Jianhong Huo**: formal analysis, project administration, validation.

## Conflicts of Interest

The authors declare no conflicts of interest.

### Peer Review

The peer review history for this article is available at https://publons.com/publon/10.1002/brb3.70188.

## Supporting information



Supporting Information.

Supporting Information.

## Data Availability

The original contributions presented in the study can be found in the article. Further queries should be directed to the corresponding author.

## References

[brb370188-bib-0001] Ahmed, Z. , and N. Tabet . 2015. “Thyroid Dysfunction and Stroke: A Review.” Journal of Stroke and Cerebrovascular Diseases 24, no. 10: 2149–2159.26142257

[brb370188-bib-0002] Al Kasab, S. , C. P. Derdeyn , W. R. Guerrero , K. Limaye , A. Shaban , and H. P. Adams Jr . 2018. “Intracranial Large and Medium Artery Atherosclerotic Disease and Stroke.” Journal of Stroke and Cerebrovascular Diseases 27, no. 7: 1723–1732. 10.1016/j.jstrokecerebrovasdis.2018.02.050. 29602616

[brb370188-bib-0003] Antonijevic, N. , D. Matic , B. Beleslin , et al. 2024. “The Influence of Hyperthyroidism on the Coagulation and on the Risk of Thrombosis.” Journal of Clinical Medicine 13, no. 6: 1756. 38541980 10.3390/jcm13061756 PMC10971193

[brb370188-bib-0004] Aslan, M. , N. Cosar , H. Celik , et al. 2011. “Evaluation of Oxidative Status in Patients With Hyperthyroidism.” Endocrine 40, no. 2: 285–289. 10.1007/s12020-011-9472-3. 21519910

[brb370188-bib-0005] Bano, A. , L. Chaker , M. P. M. de Maat , et al. 2019. “Thyroid Function and Cardiovascular Disease: The Mediating Role of Coagulation Factors.” Journal of Clinical Endocrinology and Metabolism 104: 3203–3212. 30938758 10.1210/jc.2019-00072

[brb370188-bib-0006] Bi, Y. , T. Feng , M. Li , et al. 2012. “Thyroid Function and Risk of Ischemic Stroke: A Meta‐Analysis of Prospective Studies.” Stroke 43, no. 11: 2888–2894.

[brb370188-bib-0007] Bowden, J. , G. M. F. Del , C. Minelli , et al. 2019. “Improving the Accuracy of Two‐Sample Summary‐Data Mendelian Randomization. Moving Beyond the NOME Assumption.” International Journal of Epidemiology 48: 728–742. 30561657 10.1093/ije/dyy258 PMC6659376

[brb370188-bib-0008] Burgess, S. , A. Butterworth , and S. G. Thompson . 2013. “Mendelian Randomization Analysis With Multiple Genetic Variants Using Summarized Data.” Genetic Epidemiology 37: 658–665. 10.1002/gepi.21758. 24114802 PMC4377079

[brb370188-bib-0009] Burgess, S. , G. D. Smith , N. M. Davies , et al. 2019. “Guidelines for Performing Mendelian Randomization Investigations.” Wellcome Open Research 4: 186. 32760811 10.12688/wellcomeopenres.15555.1 PMC7384151

[brb370188-bib-0010] Burgess, S. , D. S. Small , and S. G. Thompson . 2017. “A Review of Instrumental Variable Estimators for Mendelian Randomization.” Statistical Methods in Medical Research 26, no. 5: 2333–2355. 26282889 10.1177/0962280215597579 PMC5642006

[brb370188-bib-0011] Burgess, S. , S. G. Thompson , and CRP CHD Genetics Collaboration. 2011. “Avoiding Bias From Weak Instruments in Mendelian Randomization Studies.” International Journal of Epidemiology 40, no. 3: 755–764. 21414999 10.1093/ije/dyr036

[brb370188-bib-0012] Burgess, S. , N. J. Timpson , S. Ebrahim , and G. Davey Smith . 2015. “Mendelian Randomization: Where Are We Now and Where Are We Going?” International Journal of Epidemiology 44, no. 2: 379–388. 26085674 10.1093/ije/dyv108

[brb370188-bib-0013] Chaker, L. , C. Baumgartner , M. A. Ikram , et al. 2014. “Subclinical Thyroid Dysfunction and the Risk of Stroke: A Systematic Review and Meta‐Analysis.” European Journal of Epidemiology 29, no. 11: 791–800. 25179793 10.1007/s10654-014-9946-8

[brb370188-bib-0014] Chaker, L. , D. S. Cooper , J. P. Walsh , and R. P. Peeters . 2024. “Hyperthyroidism.” Lancet 403, no. 10428: 768–780. 10.1016/S0140-6736(23)02016-0. 38278171

[brb370188-bib-0015] Chen, X. , X. Hong , W. Gao , et al. 2022. “Causal Relationship Between Physical Activity, Leisure Sedentary Behaviors and COVID‐19 Risk: A Mendelian Randomization Study.” Journal of Translational Medicine 20: 216. 35562752 10.1186/s12967-022-03407-6 PMC9100292

[brb370188-bib-0016] Cheng, F. , A. O. Luk , M. Shi , et al. 2022. “Shortened Leukocyte Telomere Length Is Associated With Glycemic Progression in Type 2 Diabetes. A Prospective and Mendelian Randomization Analysis.” Diabetes Care 45: 701–709. 35085380 10.2337/dc21-1609 PMC8918237

[brb370188-bib-0017] Davey Smith, G. , and G. Hemani . 2014. “Mendelian Randomization: Genetic Anchors for Causal Inference in Epidemiological Studies.” Human Molecular Genetics 23: R89–98. 25064373 10.1093/hmg/ddu328 PMC4170722

[brb370188-bib-0018] Chaker, L. , C. Baumgartner , W. P. den Elzen , et al. 2016. “Thyroid Function Within the Reference Range and the Risk of Stroke: An Individual Participant Data Analysis.” Journal of Clinical Endocrinology and Metabolism 101, no. 11: 4270–4282. 10.1210/jc.2016-2255. 27603906 PMC5095234

[brb370188-bib-0019] Ellekjaer, E. F. , T. B. Wyller , J. M. Sverre , and J. Holmen . 1992. “Lifestyle Factors and Risk of Cerebral Infarction.” Stroke 23, no. 6: 829–834. 1595100 10.1161/01.str.23.6.829

[brb370188-bib-0020] Ellervik, C. , L. Boulakh , A. Teumer , et al. 2024. “Thyroid Function, Diabetes, and Common Age‐Related Eye Diseases: A Mendelian Randomization Study.” Thyroid 34, no. 11: 1414–1423. 39283829 10.1089/thy.2024.0257 PMC11958925

[brb370188-bib-0021] Freuer, D. , J. Linseisen , and C. Meisinger . 2022. “Association Between Inflammatory Bowel Disease and Both Psoriasis and Psoriatic Arthritis: A Bidirectional 2‐Sample Mendelian Randomization Study.” JAMA Dermatology 158, no. 11: 1262–1268. 36103169 10.1001/jamadermatol.2022.3682 PMC9475439

[brb370188-bib-0022] Grama, S. , I. Willcocks , J. J. Hubert , et al. 2020. “Polygenic Risk for Schizophrenia and Subcortical Brain Anatomy in the UK Biobank Cohort.” Translational Psychiatry 10, no. 1: 309. 32908133 10.1038/s41398-020-00940-0 PMC7481214

[brb370188-bib-0023] Hartwig, F. P. , S. G. Davey , and J. Bowden . 2017. “Robust Inference in Summary Data Mendelian Randomization via the Zero Modal Pleiotropy Assumption.” International Journal of Epidemiology 46: 1985–1998. 29040600 10.1093/ije/dyx102 PMC5837715

[brb370188-bib-0024] Hemani, G. , J. Zheng , B. Elsworth , et al. 2018. “The MR‐Base Platform Supports Systematic Causal Inference Across the Human Phenome.” Elife 7: e34408. 29846171 10.7554/eLife.34408 PMC5976434

[brb370188-bib-0025] Inoue Kosuke, G. , L. Rong , L. Martin , et al. 2023. “Iodine‐Induced Hyperthyroidism and Long‐Term Risks of Incident Atrial Fibrillation and Flutter.” Clinics in Endocrinology and Metabolism 108: e956–e962. 10.1210/clinem/dgad250 PMC10584637 37146179

[brb370188-bib-0026] Kim, H. J. , T. Kang , M. J. Kang , H. S. Ahn , and S. Y. Sohn . 2020. “Incidence and Mortality of Myocardial Infarction and Stroke in Patients With Hyperthyroidism: A Nationwide Cohort Study in Korea.” Thyroid 30, no. 7: 955–965. 32093587 10.1089/thy.2019.0543

[brb370188-bib-0027] Lo, E. H. , T. Dalkara , and M. A. Moskowitz . 2003. “Mechanisms, Challenges and Opportunities in Stroke.” Nature Reviews Neuroscience 4, no. 5: 399–415. 12728267 10.1038/nrn1106

[brb370188-bib-0028] Mokhtari, T. , M. Akbari , F. Malek , et al. 2017. “Improvement of Memory and Learning by Intracerebroventricular Microinjection Fof T3 in Rat Model of Ischemic Brain Stroke Mediated by Upregulation of BDNF and GDNF in CA1 Hippocampal Region.” DARU Journal of Pharmaceutical Sciences 25, no. 1: 4. 10.1186/s40199-017-0169-x. 28202057 PMC5312580

[brb370188-bib-0029] Ohba, S. , T. Nakagawa , and H. Murakami . 2011. “Concurrent Graves' Disease and Intracranial Arterial Stenosis/Occlusion: Special Considerations Regarding the State of Thyroid Function, Etiology, and Treatment.” Neurosurgical Review 34, no. 3: 297–304. 21424208 10.1007/s10143-011-0311-z

[brb370188-bib-0030] Pappan, N. , M. T. Ud Din , D. Venkat , P. Wedgeworth , and S. Fu . 2022. “Screening for Thyroid Disorders Among Resistant Hypertension Patients: Are We Doing Enough?” Clinical Medicine & Research 20: 70–73. 34996821 10.3121/cmr.2021.1676 PMC9242733

[brb370188-bib-0031] Pierce, B. L. , H. Ahsan , and T. J. Vanderweele . 2011. “Power and Instrument Strength Requirements for Mendelian Randomization Studies Using Multiple Genetic Variants.” International Journal of Epidemiology 40: 740–752. 20813862 10.1093/ije/dyq151 PMC3147064

[brb370188-bib-0032] Rutten‐Jacobs, L. C. , S. C. Larsson , R. Malik , et al. 2018. “Genetic Risk, Incident Stroke, and the Benefits of Adhering to a Healthy Lifestyle: Cohort Study of 306 473 UK Biobank Participants.” BMJ 363: k4168. 30355576 10.1136/bmj.k4168 PMC6199557

[brb370188-bib-0033] Sekula, P. , M. F. Del Greco , C. Pattaro , and A. Köttgen . 2016. “Mendelian Randomization as an Approach to Assess Causality Using Observational Data.” Journal of the American Society of Nephrology 27: 3253–3265. 27486138 10.1681/ASN.2016010098 PMC5084898

[brb370188-bib-0034] Sheu, J. J. , J. H. Kang , H. C. Lin , and H. C. Lin . 2010. “Hyperthyroidism and Risk of Ischemic Stroke in Young Adults: A 5‐Year Follow‐Up Study.” Stroke 41, no. 5: 961–966. 20360542 10.1161/STROKEAHA.109.577742

[brb370188-bib-0035] Skrivankova, V. W. , R. C. Richmond , B. A. R. Woolf , et al. 2021. “Strengthening the Reporting of Observational Studies in Epidemiology Using Mendelian Randomization (STROBE‐MR): Explanation and Elaboration.” BMJ 375: n2233. 34702754 10.1136/bmj.n2233 PMC8546498

[brb370188-bib-0036] Smith, G. D. , and S. Ebrahim . 2004. “Mendelian Randomization: Prospects, Potentials, and Limitations.” International Journal of Epidemiology 33: 30–42. 15075143 10.1093/ije/dyh132

[brb370188-bib-0037] Smith, G. D. , D. A. Lawlor , R. Harbord , N. Timpson , I. Day , and S. Ebrahim . 2007. “Clustered Environments and Randomized Genes: A Fundamental Distinction Between Conventional and Genetic Epidemiology.” PLoS Medicine 4: e352. 18076282 10.1371/journal.pmed.0040352 PMC2121108

[brb370188-bib-0038] Sproviero, W. , L. Winchester , D. Newby , et al. 2021. “High Blood Pressure and Risk of Dementia: A Two‐Sample Mendelian Randomization Study in the UK Biobank.” Biological Psychiatry 89: 817–824. 33766239 10.1016/j.biopsych.2020.12.015

[brb370188-bib-0039] Surks, M. I. , E. Ortiz , G. H. Daniels , et al. 2004. “Subclinical Thyroid Disease: Scientific Review and Guidelines for Diagnosis and Management.” Journal of the American Medical Association 291, no. 2: 228–238. 14722150 10.1001/jama.291.2.228

[brb370188-bib-0040] Taylor, P. N. , M. M. Medici , A. Hubalewska‐Dydejczyk , and K. Boelaert . 2024. “Hypothyroidism.” Lancet 404, no. 10460: 1347–1364. 10.1016/S0140-6736(24)01614-3. 39368843

[brb370188-bib-0041] Thompson, S. G. , J. Kienast , S. D. Pyke , F. Haverkate , and J. C. van de Loo . 1995. “Hemostatic Factors and the Risk of Myocardial Infarction or Sudden Death in Patients With Angina Pectoris. European Concerted Action on Thrombosis and Disabilities Angina Pectoris Study Group.” New England Journal of Medicine 332, no. 10: 635–641. 7845427 10.1056/NEJM199503093321003

[brb370188-bib-0042] Venkatesh, S. S. , T. Ferreira , S. Benonisdottir , et al. 2022. “Obesity and Risk of Female Reproductive Conditions: A Mendelian Randomization Study.” PLoS Medicine 19: e1003679. 35104295 10.1371/journal.pmed.1003679 PMC8806071

[brb370188-bib-0043] Wang, T. , X. Wang , J. Wu , and X. Li . 2024. “Causal Relationship Between Thyroid Dysfunction and Ovarian Cancer: A Two‐Sample Mendelian Randomization Study.” BMC Cancer 24, no. 1: 629. 38783224 10.1186/s12885-024-12385-5 PMC11112802

[brb370188-bib-0044] Wang, W. , B. Jiang , H. Sun , et al. 2017. “Prevalence, Incidence, and Mortality of Stroke in China: Results From a Nationwide Population‐Based Survey of 480,687 Adults.” Circulation 135, no. 8: 759–771. 28052979 10.1161/CIRCULATIONAHA.116.025250

[brb370188-bib-0045] Weihs, A. , L. Chaker , T. C. Martin , et al. 2023. “Epigenome‐Wide Association Study Reveals CpG Sites Associated With Thyroid Function and Regulatory Effects on KLF9.” Thyroid 33, no. 3: 301–311. 36719767 10.1089/thy.2022.0373 PMC10024591

[brb370188-bib-0046] Zheng, J. , D. Baird , M. C. Borges , et al. 2017. “Recent Developments in Mendelian Randomization Studies.” Current Epidemiology Reports 4: 330–345. 29226067 10.1007/s40471-017-0128-6 PMC5711966

