# Supplementary material for: Mendelian Randomization Study Investigating the Causal Relationship Between Thyroid Dysfunction and Cerebral Infarction
Source: Brain Behav. 2024 Dec 11;14(12):e70188. doi: 10.1002/brb3.70188 (PMC11635123; doi:10.1002/brb3.70188)

Figure S1: Forest map of MR analysis. (A)hyperthyroidism and cerebral infarction;(B)hypothyroidism and cerebral infarction; (C)FT4 and cerebral infarction; (D)TSH and cerebral infarction


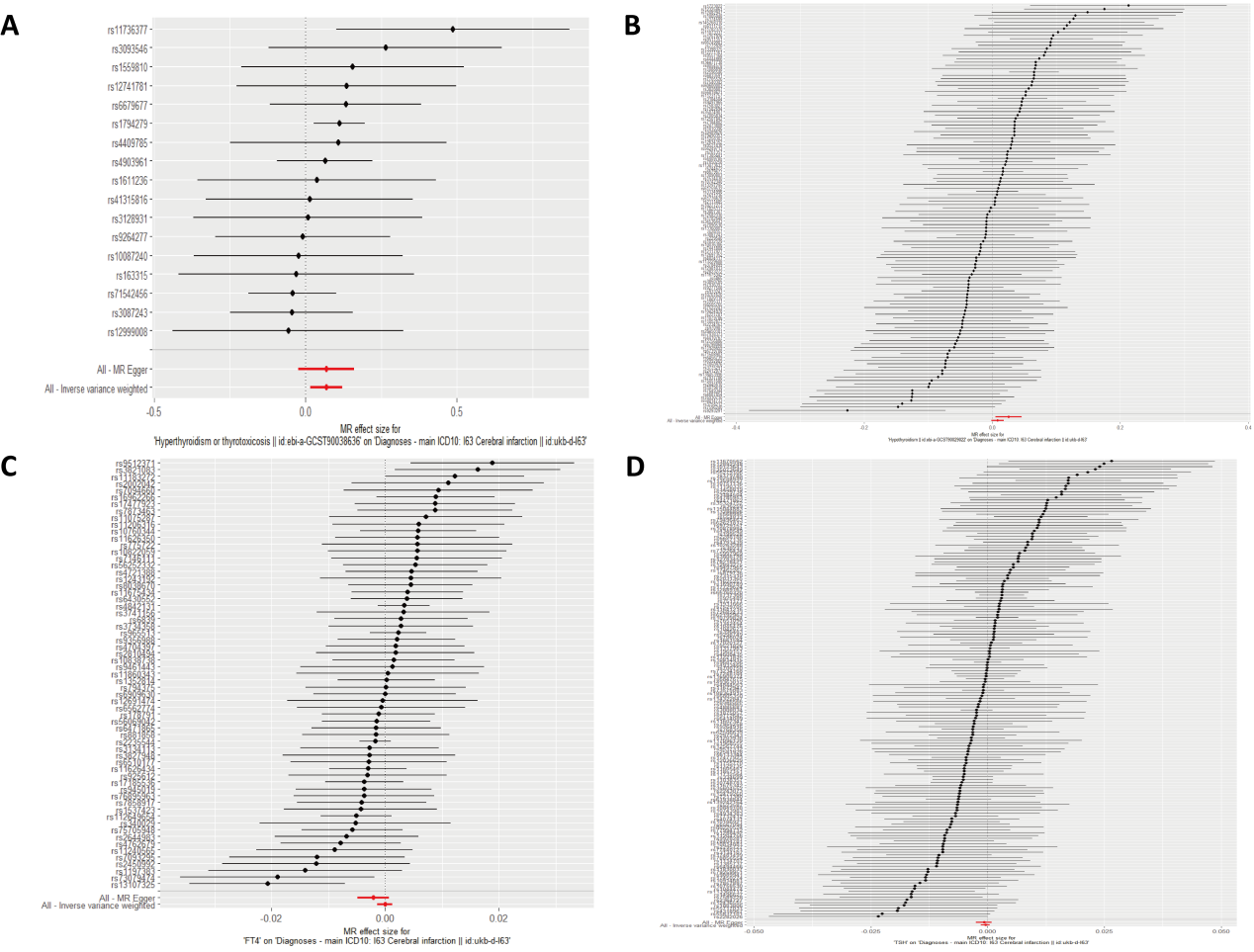


Figure S2: Funnel map of MR analysis. (A)hyperthyroidism and cerebral infarction;(B)hypothyroidism and cerebral infarction; (C)FT4 and cerebral infarction; (D)TSH and cerebral infarction


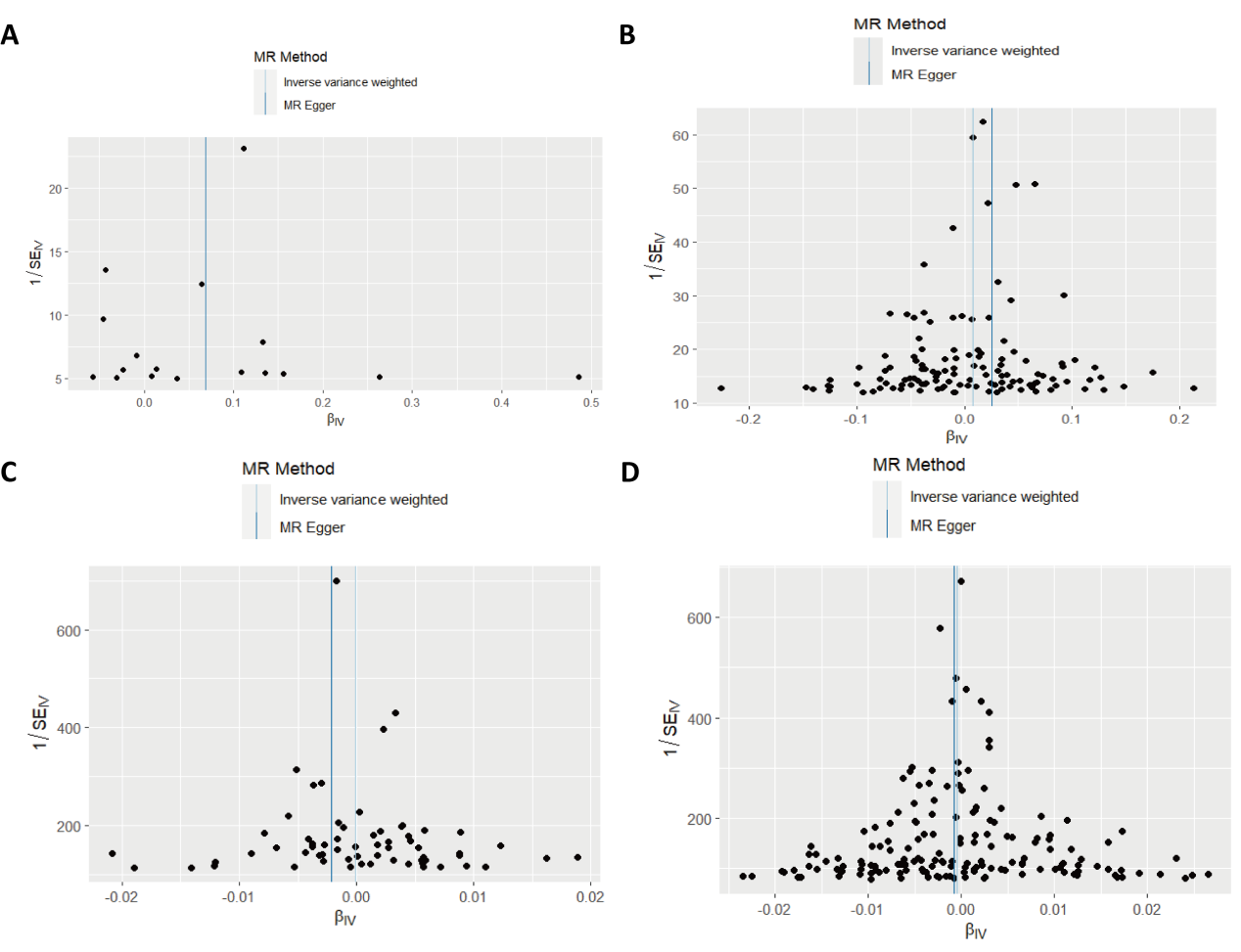


Figure S3: Leave-one-out map of MR analysis. (A)hyperthyroidism and cerebral infarction;(B)hypothyroidism and cerebral infarction; (C)FT4 and cerebral infarction; (D)TSH and cerebral infarction


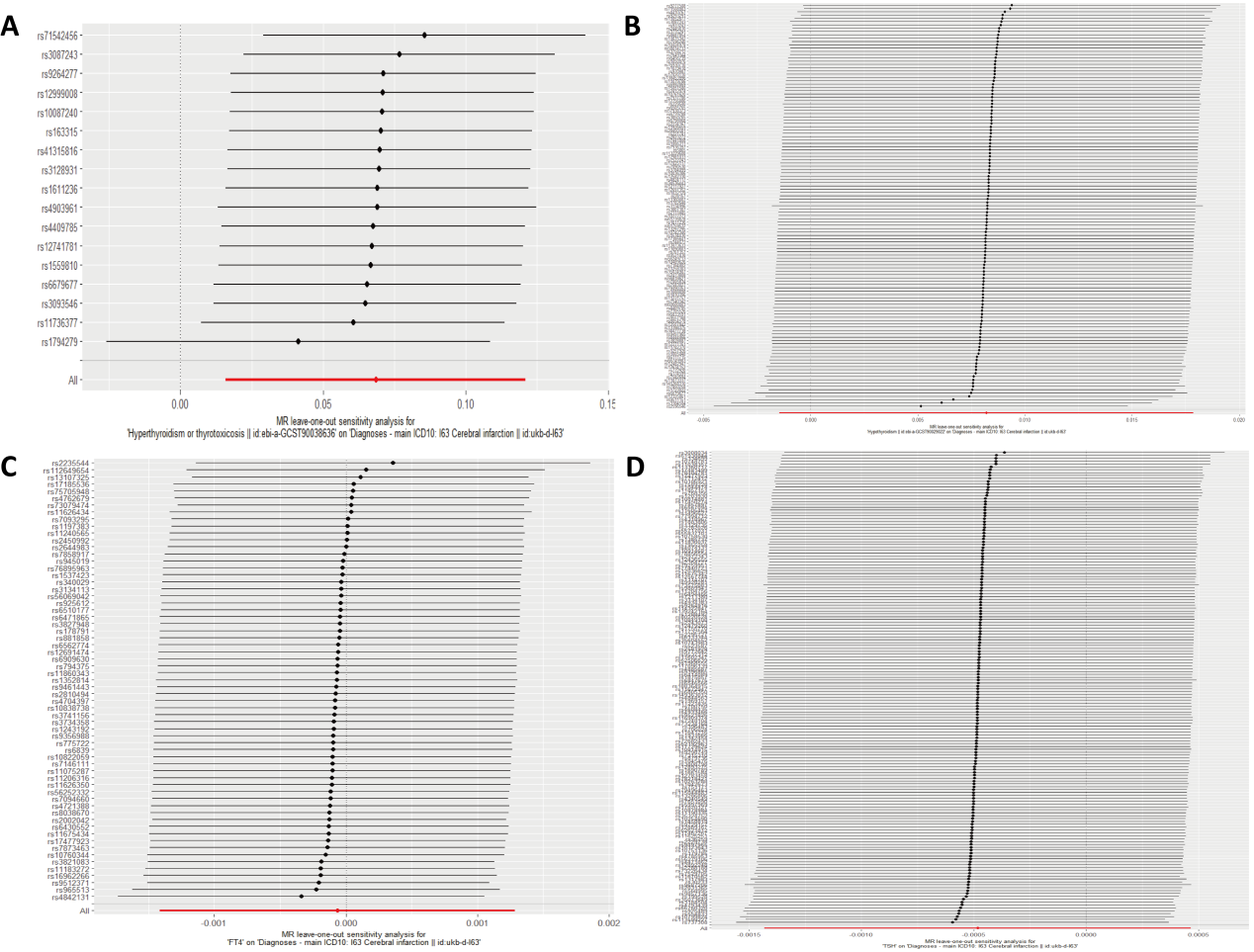


Figure S4: Forest map of reverse MR analysis. (A)hyperthyroidism and cerebral infarction;(B)hypothyroidism and cerebral infarction; (C)FT4 and cerebral infarction; (D)TSH and cerebral infarction


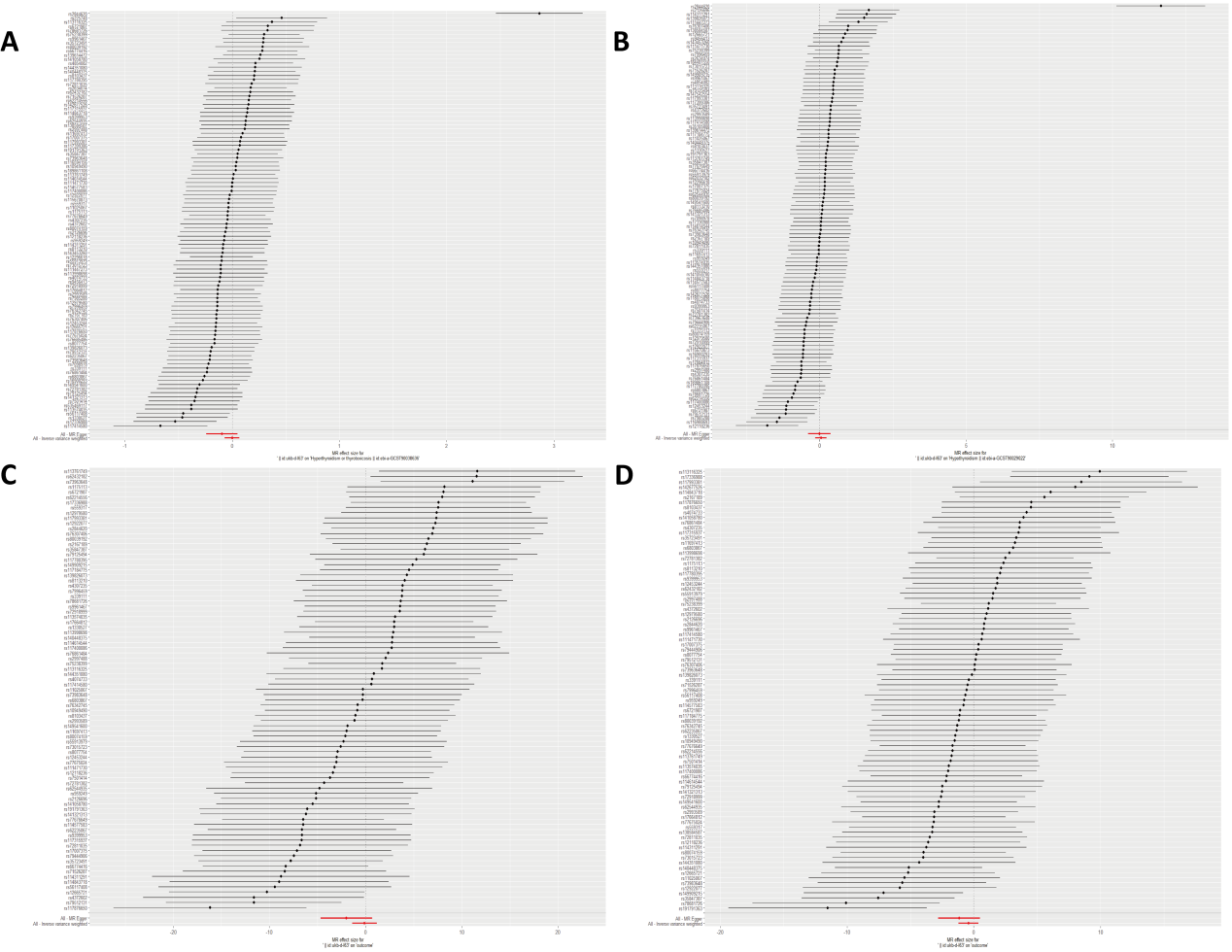


Figure S5: Funnel map of reverse MR analysis. (A)hyperthyroidism and cerebral infarction;(B)hypothyroidism and cerebral infarction; (C)FT4 and cerebral infarction; (D)TSH and cerebral infarction


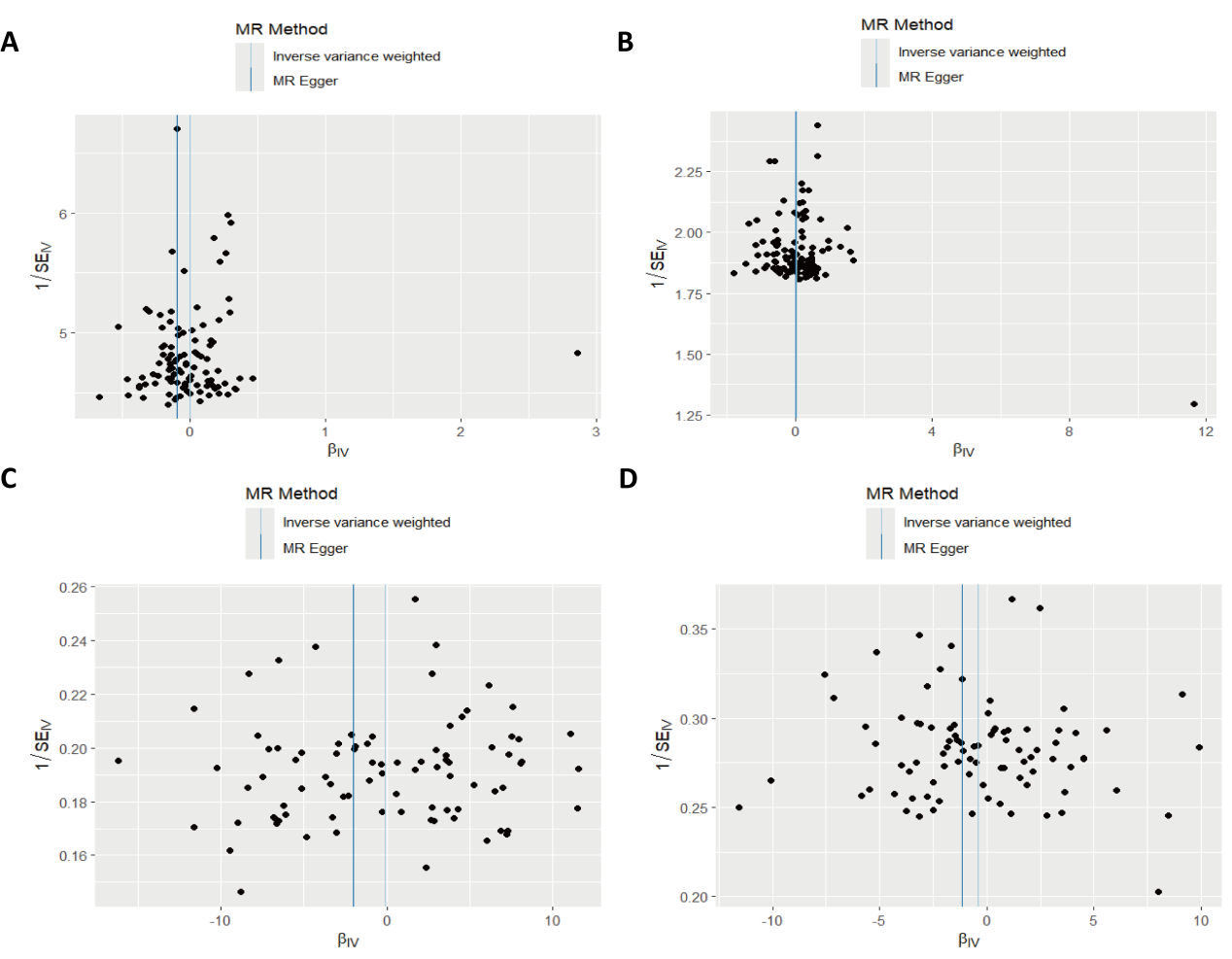


Figure S6: Leave-one-out map of reverse MR analysis. (A)hyperthyroidism and cerebral infarction;(B)hypothyroidism and cerebral infarction; (C)FT4 and cerebral infarction; (D)TSH and cerebral infarction


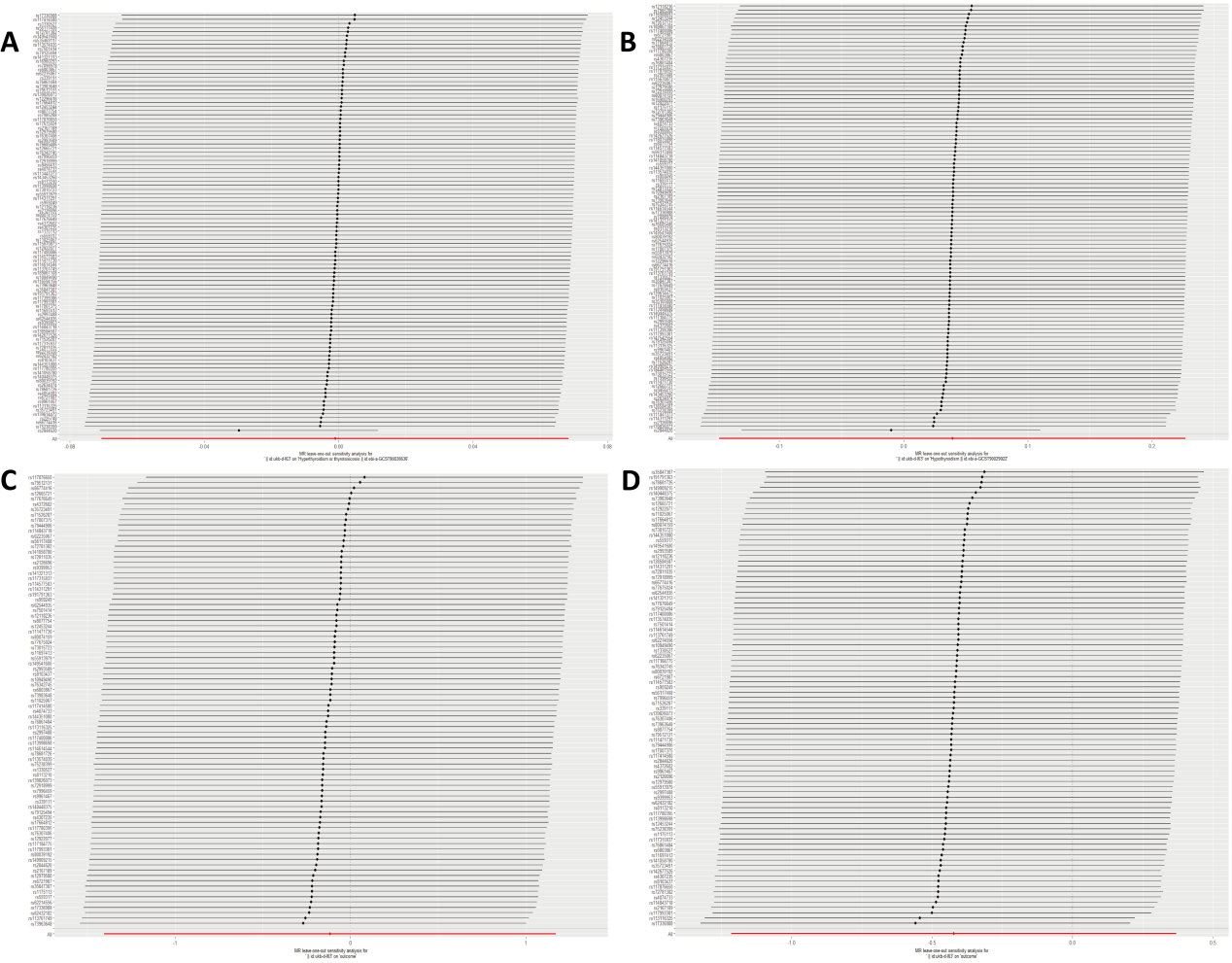

Supplement: Supplementary file 1 — Supporting Information. [file BRB3-14-e70188-s001.docx]
